# Stability of the Structural and Transport Characteristics of (ZrO_2_)_0.99−x_(Sc_2_O_3_)_x_(R_2_O_3_)_0.01_ (R–Yb, Y, Tb, Gd) Electrolytic Membranes to High-Temperature Exposure

**DOI:** 10.3390/membranes13030312

**Published:** 2023-03-09

**Authors:** Dmitrii Agarkov, Mikhail Borik, Galina Korableva, Alexey Kulebyakin, Irina Kuritsyna, Nataliya Larina, Elena Lomonova, Filipp Milovich, Valentina Myzina, Polina Ryabochkina, Nataliya Tabachkova, Tatyana Volkova, Denis Zakharov

**Affiliations:** 1 Osipyan Institute of Solid State Physics RAS, Academician Osipyan Str., 2, 142432 Chernogolovka, Moscow District, Russia; 2 Moscow Institute of Physics and Technology, Instituskiy per., 9, 141701 Doloprudny, Moscow District, Russia; 3 Prokhorov General Physics Institute of Russian Academy of Sciences, Vavilova Street, 38, 119991 Moscow, Russia; 4 Institute of High Technologies and New Materials, National Research Ogarev Mordovia State University, Bolshevistskaya Street, 68, 430005 Saransk, Russia; 5 Department of Materials Science, Moscow Polytechnic University, Bolshaya Semyonovskaya Street, 38, 107023 Moscow, Russia; 6 Department of Materials Science of Semiconductors and Dielectrics, National University of Science and Technology «MISIS», Leninskiy Prospect, 4, 119049 Moscow, Russia

**Keywords:** zirconia, SOFC, single crystal, structure, luminescence spectra

## Abstract

The effect of long-term high-temperature annealing on the phase composition, local crystal structure, and oxygen-ion conductivity of SOFC membranes based on zirconium dioxide solid solutions was studied. Crystals with the composition of (ZrO_2_)_0.99−x_(Sc_2_O_3_)_x_(R_2_O_3_)_0.01_ (where x = 0.08–0.1; R-Yb, Y, Tb, Gd) were obtained by the method of directed melt crystallization in a cold crucible. The crystals were annealed in air at a temperature of 1000 °C for 400 h. The phase analysis of the crystals before and after annealing was studied by X-ray diffractometry and Raman spectroscopy. The study of the ionic conductivity of the crystals was carried out by the method of impedance spectroscopy in the temperature range 400–900 °C. It has been shown that when various rare earth cations (Yb, Y, Tb, and Gd) are used, the maximum conductivity is observed for the compositions (ZrO_2_)_0.91_(Sc_2_O_3_)_0.08_(Yb_2_O_3_)_0.01_, (ZrO_2_)_0.89_(Sc_2_O_3_)_0.1_(Y_2_O_3_)_0.01_, (ZrO_2_)_0.90_(Sc_2_O_3_)_0.09_(Tb_2_O_3_)_0.01_, and (ZrO_2_)_0.89_(Sc_2_O_3_)_0.1_(Gd_2_O_3_)_0.01_. At the same time, these crystals have a highly symmetrical pseudocubic structure, which is retained even after crystal annealing. At comparable concentrations of Sc_2_O_3_, the conductivity of crystals decreases with an increase in the ionic radius of the rare earth cation. The high-temperature degradation of the conductivity is also discussed depending on the type of rare earth oxide and the concentration of scandium oxide.

## 1. Introduction

The use of fossil fuel as a source of energy has destructive environmental effects and impacts on the climate. In addition, fossil fuel has limited resources. Therefore, much attention is paid to the development of alternative renewable and stable sources of energy. Fuel cell technologies are one of the most efficient technologies for power generation, as it is a flexible fuel choice, having low CO_2_ emissions, being environment friendly, and having a potentially long lifetime of 40,000–80,000 h [1,2,3]. Solid oxide fuel cells (SOFCs) directly convert the chemical energy stored in fuel into electrical energy through electrochemical reactions. The creation of power sources based on electrochemical generators with SOFCs plays an important role in providing an autonomous power supply for industrial infrastructure facilities, transport, and domestic needs of the population [4,5,6].

Current and widely used materials for the manufacture of electrolytic membranes are solid solutions based on ZrO_2_ [7,8,9]. In recent years, much attention has been paid to the study of scandium oxide-stabilized zirconia, which has the highest conductivity. However, this material is subject to degradation of the electrical conductivity during long-term operation at high operating temperatures. To increase the stability of the structure and transport characteristics, additional codoping oxides are introduced into the composition of ZrO_2_-Sc_2_O_3_ solid solutions [10,11,12,13,14,15].

A number of works are devoted to the study of the high-temperature degradation of ZrO_2_-Sc_2_O_3_ solid solutions [16,17,18,19,20,21]. The following mechanisms were considered reasons for degradation: phase changes leading to the formation of inclusions of a low-conductivity tetragonal phase in a cubic matrix [16]; the formation of various associates of defects [17]; and local ordering of oxygen ions and oxygen vacancies [18].

The study of the degradation of ZrO_2_-Sc_2_O_3_ solid solutions additionally doped with oxides of rare earth elements is considered in [9,12,14,22]. It was shown in [14] that during the aging of the (ZrO_2_)_0.90_(Sc_2_O_3_)_0.09_(Yb_2_O_3_)_0.01_ and (ZrO_2_)_0.90_(Sc_2_O_3_)_0.09_(Gd_2_O_3_)_0.01_ samples, the volume conductivity decreased by 33.7 and 15.2%, respectively. The observed degradation of conductivity was associated with the formation of a tetragonal phase. An increase in the concentration of the codoping impurity to 2 mol% prevents the precipitation of the tetragonal phase; however, in this case, a slight decrease in the conductivity of the samples was observed as well.

Thus, the thermal stability of ZrO_2_-Sc_2_O_3_ solid solutions additionally doped with oxides of rare earth elements depends on the type of stabilizing and codoping impurities and their concentration.

In this work, we studied the effect of long-term high-temperature annealing in air on the stability of the phase composition, local structure, and transport characteristics of (ZrO_2_)_0.99−x_(Sc_2_O_3_)_x_(R_2_O_3_)_0.01_ single crystals, where R = Y, Gd, Tb, and Yb and x = 0.08–0.10. In addition, an attempt was made to establish a possible correlation between the radius of the codoping oxide cation and the structural and transport characteristics of solid solutions based on ZrO_2_-Sc_2_O_3_.

The values of the ionic radii of the codoping cations Gd^3+^, Tb^3+^, Tb^4+^, Y^3+^, and Yb^3+^ for the oxygen coordination number 8 are 1.053, 1.04, 0.88, 1.019, and 0.985 Å, respectively [23].

## 2. Materials and Methods

Crystals of solid solutions (ZrO_2_)_0.99−x_(Sc_2_O_3_)_x_(R_2_O_3_)_0.01_ (R—Yb, Y, Tb, Gd) were grown by the method of directed melt crystallization [24]. Some crystals were additionally doped with europium oxide, which was used as a spectroscopic probe. The initial powders were melted in a water-cooled copper crucible with a diameter of 130 mm. A high-frequency generator with a power of 100 kW and a frequency of 5.28 MHz was used as a heating source. Directed crystallization of the melt was carried out by lowering the crucible relative to the induction coil at a speed of 10 mm/h. The mass of the melt was 5 kg. Powders of oxides of zirconium, scandium, and rare earth elements with a purity of at least 99.99% were used as initial reagents. As a result, a crystallized ingot was obtained, consisting of individual columnar crystals, and the separation of the ingot into individual crystals was carried out mechanically. Typical crystal sizes were 20–30 mm in diameter and 40–50 mm in height. Heat treatment of the crystals was carried out in a Nabertherm HT04/16 (Germany) high-temperature resistance furnace at a temperature of 1000 °C in air for 400 h. The samples for research in the form of plane-parallel plates were cut out from the central part of the crystals.

Phase analysis was performed by X-ray diffraction on a Bruker D8 diffractometer using CuKα radiation with a standard method used for single crystals. The specimens were in the form of plates cut perpendicular to the 〈001〉 direction. For the phase analysis, planes with different indexes and different phases were set to the reflecting position using the three-circle goniometer. The lattice parameters were determined from the diffraction maxima at large angles of 2θ ≈ 130°. The phase analysis of the crystals was also carried out by Raman spectroscopy (RS) [25,26,27,28] using a laser with a wavelength of 633 nm as an excitation source. Microstructural studies were carried out on a Carl Zeiss Axio Imager Z2 (Germany) optical microscope in polarized transmitted light on plane-parallel plates with a thickness of 1.5 mm.

A study of the local structure of the crystals was carried out by optical spectroscopy. The luminescence spectra of the Eu^3+^ ions were recorded using a Horiba FHR 1000 (Japan) spectrometer. The Eu^3+^ ions were excited to the 5D1 level by the second harmonic of radiation from a YVO4:Nd (λexc = 532 nm) and a LiYF4:Nd (λexc = 527 nm) lasers. A Photomultiplier tube (PMT), Hamamatsu R928, was used as a radiation receiver. The registration of the luminescence spectra of the studied samples of the crystals was carried out at T = 300 K.

Studies of the conductivity of the crystals were carried out in the temperature range of 400–900 °C using a Solartron SI 1260 frequency-response analyzer in the frequency range of 1 Hz–5 MHz with an alternating current signal amplitude of 24 mV. The measurements were carried out on plates with a size of 7 × 7 mm and thickness of 0.5 mm. To form the current contacts, platinum paste was applied to opposite sides of the samples, which was fired at a temperature of 900 °C for 1 h in air. The impedance spectra were processed using the ZView program (ver. 2.8). The specific conductivity of the crystals was calculated from the data obtained by processing the impedance spectra, taking into account the geometric dimensions of the samples. The relative error of the conductivity measurements was 0.5–1%.

## 3. Results and Dictation

Four series of crystals were grown, and their compositions, short designations, phase composition, and a description of the appearance are provided in Table 1. The data given in Table 1 on the phase composition were obtained by X-ray diffractometry.

**Figure 1:**
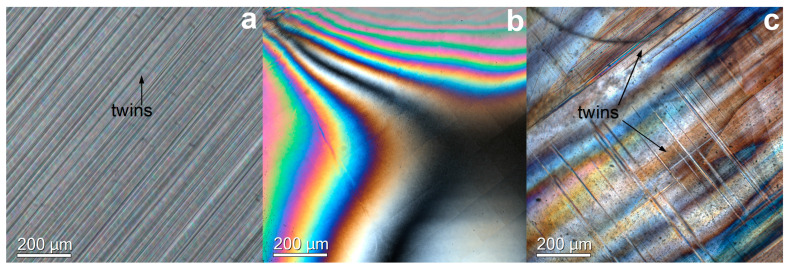
Typical optical images characteristics of tetragonal (**a**); cubic (**b**); rhombohedral (**c**).

As one can see from Table 1, the transparent crystals are single-phase single crystals with a cubic fluorite structure. The presence of completely or partially muddy regions of crystals is associated with the formation of a twin structure characteristic of a tetragonal or rhombohedral phase. Figure 1 shows typical images of the microstructure of the crystals corresponding to different phases, obtained in transmitted polarized light.

As follows from Figure 1, the tetragonal and rhombohedral phases contain twins. The average size of the twins observed by the method of optical microscopy was ~5 and 50 microns for the tetragonal and optical structure, respectively. Thus, the size of the twins of the rhombohedral phase was much larger than that of the tetragonal phase. The crystals with a cubic structure did not contain visible defects. In Figure 1b,c one can see the interference patterns due to the presence of stresses in the samples. It should be noted that annealing did not lead to stress relief.

The grown crystals containing 8 mol% scandium oxide were a tetragonal modification of zirconia in the entire volume of crystals, regardless of the type of stabilizing oxide. The phase composition of the crystals containing 9 mol% Sc_2_O_3_ and additionally stabilized with 1 mol% R_2_O_3_ (where R is Yb, Tb, Y, and Gd) depended on the type of R_2_O_3_. For example, in crystals additionally doped with Yb_2_O_3_ or Tb_2_O_3_, the cubic phase stabilized throughout the entire volume of the crystals. The crystals additionally doped with Y_2_O_3_ or Gd_2_O_3_ were two-phase and represented a mixture of tetragonal and cubic modifications of ZrO_2_. In this case, the cubic phase was mainly formed in the lower part, and the tetragonal phase in the upper part of the crystal. The crystals containing 10 mol% Sc_2_O_3_ had a cubic fluorite structure, except for the crystals codoped with Yb_2_O_3_. These crystals were two-phase and contained cubic and rhombohedral phases. Thus, for the studied compositions, the minimum concentration of Sc_2_O_3_ required to stabilize the cubic phase was 9 mol% in the case of Yb_2_O_3_ or Tb_2_O_3.5_ codoping and 10 mol% for Y_2_O_3_ or Gd_2_O_3_.

After annealing, all single-phase crystals (both tetragonal and cubic) remained single phase. The annealing of the two-phase crystals led either to a decrease in the amount of the cubic phase (9Sc1YSZ) or to its complete transformation into a tetragonal (9Sc1GdSZ) or rhombohedral (10Sc1YbSZ) phase.

**Figure 2:**
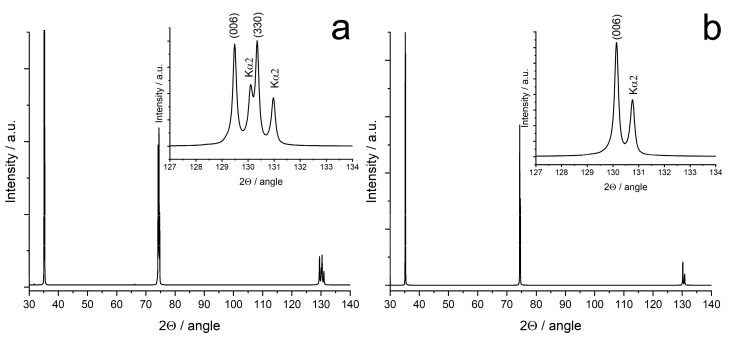
Diffractograms of 8Sc1GdSZ (**a**) and 10Sc1GdSZ (**b**) crystals.

As an example, Figure 2 shows a diffractogram for the tetragonal 8SC1GDSZ and cubic 10SC1GDSZ crystals. Table 2 and Table 3 list the lattice parameters of the tetragonal and cubic phases before and after crystal annealing.

A comparison of the lattice parameters of the tetragonal crystals containing 8 mol% Sc_2_O_3_ showed that the “a” and “c” crystal lattice parameters differed depending on the type of rare earth oxide cation, namely, they increased in the series Yb < Tb < Y < Gd. This pattern agrees well with an increase in the ionic radius of the stabilizing oxide cation. It is known that terbium oxide has a variable valence and can enter the ZrO_2_ crystal lattice in the form of Tb^3+^ and Tb^4+^ cations; however, it was shown in a number of works that it enters this matrix mainly in the form of Tb^3+^ [29,30,31]. This provides grounds to place Tb in the series between Yb and Y. The annealing of crystals leads to an increase in the lattice parameter “c”, while the parameter “a” practically does not change. Thus, annealing of crystals containing 8 mol% Sc_2_O_3_ led to an increase in their degree of tetragonality (c/√2a).

The lattice parameter of cubic crystals (Table 3) before annealing also depends on the type of rare earth oxide and also increases with an increase in the ionic radius of the cation in the series Yb < Tb < Y < Gd. The annealing of cubic crystals does not lead to a change in the lattice parameters, except for crystals containing terbium oxide, for which a slight decrease in the lattice parameter of the cubic phase is observed, which may be due to a change in the degree of oxidation of some of the Tb^3+^ ions to Tb^4+^.

**Figure 3:**
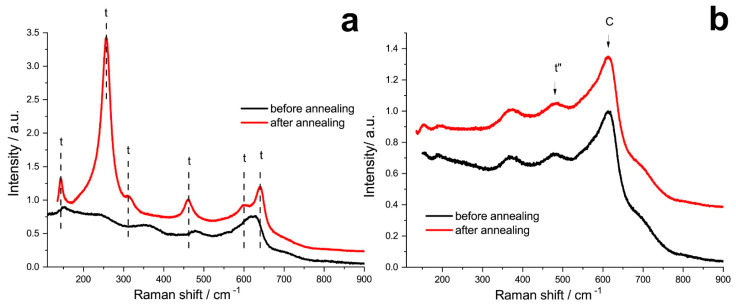
Raman spectra of 8Sc1GdSZ (**a**) and 10Sc1GdSZ (**b**) crystals before and after annealing.

The phase composition of the crystals was also studied by the Raman spectroscopy technique, which makes it possible to record changes occurring in the anionic sublattice of crystals. Figure 3 shows the typical spectra of crystals with tetragonal and cubic structures, which correspond to previously published data [32,33,34]. In the Raman spectra of 8Sc1GdSZ crystals after annealing, some bands shift to shorter wavelengths (152.3, 249.2, and 479.3 cm^−1^), which may correspond to the shortening of the O–Zr–O bond. At the same time, another group of vibration modes softens (592.8, 619.2, and 636.0 cm^−1^), in other words, leads to an increase in the values of wave numbers (601.3, 628.3, and 641.9 cm^−1^), which corresponds to the transition to the tetragonal system and, possibly, to the lengthening of the bond Zr-OI. After annealing, the ~316 and 257 cm^−1^ lines appeared, which corresponded to Zr-OI/OII and Zr-OII vibrations in the ZrO_2_ tetragonal modification. An increase in the intensity of the bands (~145, 260, 315, 475, 598, and 640 cm^−1^) corresponding to the tetragonal structure was also observed. Similar changes in the spectra after annealing are characteristic of all crystals containing 8 mol% Sc_2_O_3_. Perhaps this is due to the fact of an increase in the degree of tetragonality (c/√2a) of the crystals, which was observed in the study by X-ray diffractometry. In the spectra of cubic crystals, along with the bands characteristic of the cubic phase (~620 cm^−1^), there was also a band in the region of 480 cm^−1^, which is attributed to the presence of the t” phase in the crystals [35]. This phase was described to have the tetragonality *c/√2a* = 1 but pertaining to the P4_2_/nmc space symmetry group due to the shift in the oxygen atoms in the anion sublattice. Therefore, this phase can be considered as a pseudocubic (distorted fluorite structure). Weak bands in the region of ~370 cm^−1^ and ~700 cm^−1^ are associated with the formation of oxygen vacancies upon heterovalent substitution of Zr cations by trivalent cations of stabilizing oxides. The annealing did not lead to noticeable changes in the Raman spectra.

**Figure 4:**
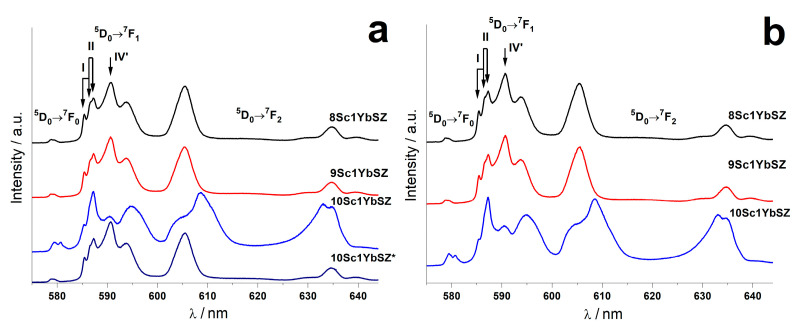
Luminescence spectra of crystals from the (ZrO_2_)_0.99−x_(Sc_2_O_3_)_x_(Yb_2_O_3_)_0.01_ series before (**a**) and after (**b**) heat treatment. (10Sc1YbSZ*—luminescence spectra for the upper part of the crystal).

The position of the oxygen vacancies relative to the cations of the stabilizing oxides and the base cation (Zr^4+^) was studied by analyzing the luminescence spectra. Figure 4, as an example, shows the luminescence spectra of crystals from the (ZrO_2_)_0.99−x_(Sc_2_O_3_)_x_(Yb_2_O_3_)_0.01_ series before and after heat treatment due to the ^5^D_0_→^7^F_0_, ^5^D_0_→^7^F_1_ and ^5^D_0_→^7^F_2_ transitions of the Eu^3+^ ions. The luminescence spectra of the other crystals from the other series were similar to those of 8Sc1YbSZ and 9Sc1YbSZ.

As one can see from the spectra, for all of the crystals under study, except for 10Sc1YbSZ, lines are observed corresponding to the optical centers of three types I, II, and IV’. A detailed identification of these centers in solid solutions based on ZrO_2_ is given in [36]. In the type I center, the oxygen vacancy is located in the first coordination sphere of the cation; in the type II center, it is located in the second coordination sphere, and in the type IV’ center the vacancy is present in more distant coordination spheres than the second one.

The luminescence spectra of the 10Sc1YbSZ crystals before the annealing differed significantly in the upper and lower parts of the crystal. For the upper part of the crystals, the luminescence spectra were a superposition of lines characteristic of the optical centers of three types I, II, and IV’ and differed little from the spectra of the other crystals. However, for the lower part of the 10Sc1YbSZ crystal, the shape of the spectrum changed, and lines appeared that are characteristic of the low-symmetry centers of Eu^3+^ ions located at the centers of six-vertex polyhedra. These optical centers are characterized by the presence of two oxygen vacancies in the first coordination sphere; this is center type III [36]. Such centers correspond to a rhombohedral crystal structure, where some of the cations have an oxygen coordination number of six. After heat treatment, the luminescence spectra of the Eu^3+^ ions characteristic of the rhombohedral phase were observed throughout the entire volume of the 10Sc1YbSZ crystal.

**Figure 5:**
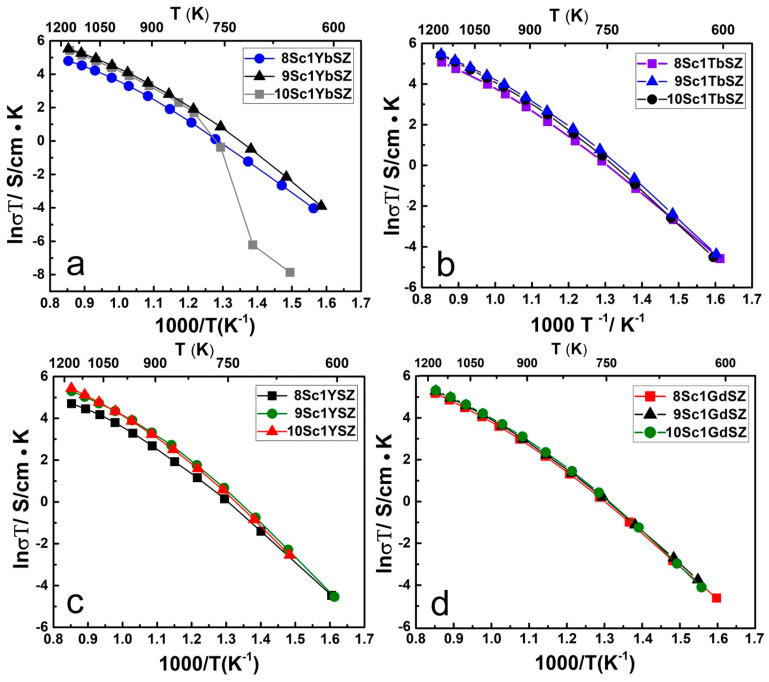
Temperature dependences of the electrical conductivity of crystals (ZrO_2_)_0.99−x_(Sc_2_O_3_)_x_(Yb_2_O_3_)_0.01_ (**a**); (ZrO_2_)_0.99−x_(Sc_2_O_3_)_x_(Tb_2_O_3_)_0.01_ (**b**); (ZrO2)_0.99−x_(Sc_2_O_3_)_x_(Y_2_O_3_)_0.01_ (**c**); (ZrO_2_)_0.99−x_(Sc_2_O_3_)_x_(Gd_2_O_3_)_0.01_ (**d**).

Figure 5 shows the temperature dependences of the electrical conductivity of four series of growth crystals in Arrhenius coordinates.

The given temperature dependences of the electrical conductivity of crystals in the Arrhenius coordinates were characterized by a noticeable curvature, which indicates different values of the activation energy in different temperature ranges. The curve of the temperature dependence of the electrical conductivity of the 10Sc1YbSZ crystal in the temperature range ~710–830 K shows a sharp inflection, which is due to the rhombohedral-cubic phase transition. The maximum values of electrical conductivity in the high-temperature region in each series of crystals were observed for the compositions 9Sc1YbSZ, 9Sc1TbSZ, 10Sc1YSZ, and 10Sc1GdSZ. Note that all these crystals are single phase and have a pseudocubic (t”) structure. Among these samples, the maximum conductivity decreases in the series 9Sc1YbSZ > 9Sc1TbSZ > 10Sc1YSZ > 10Sc1GdSZ. This sequence correlates with a change in the lattice parameter of the cubic crystals, which increases with an increase in the ionic radius of the rare earth cation.

**Figure 6:**
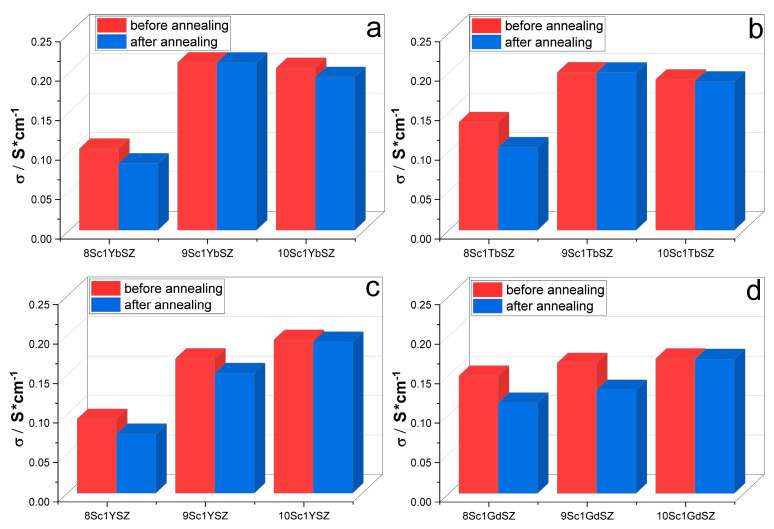
Specific electrical conductivity of crystals (ZrO_2_)_0.99−x_(Sc_2_O_3_)_x_(Yb_2_O_3_)_0.01_ (**a**); (ZrO_2_)_0.99−x_(Sc_2_O_3_)_x_(Tb_2_O_3_)_0.01_ (**b**); (ZrO_2_)_0.99−x_(Sc_2_O_3_)_x_(Y_2_O_3_)_0.01_ (**c**); (ZrO_2_)_0.99−x_(Sc_2_O_3_)_x_(Gd_2_O_3_)_0.01_ (**d**).

Figure 6 shows the electrical conductivity values at a temperature of 1173 K for the four series of crystals before and after annealing in air.

As can be seen from the diagrams, the most noticeable degradation of the electrical conductivity of crystals after annealing is observed for crystals with a tetragonal structure (8Sc1YbSZ, 8Sc1TbSZ, 8Sc1YSZ, and 8Sc1GdSZ). It can be assumed that the observed decrease in electrical conductivity is associated with an increase in the degree of crystal tetragonality upon annealing. A decrease in electrical conductivity was also observed in two-phase crystals containing a tetragonal and cubic phase (9Sc1YSZ and 9Sc1GdSZ). In this case, the observed changes are associated with a decrease in the content of the highly conductive cubic phase as a result of annealing. The annealing of single-phase pseudocubic (t” phase) crystals did not lead to noticeable changes in electrical conductivity.

## 4. Conclusions

The effect of high-temperature long-term annealing on the structural and electrotransport characteristics of electrolytic membranes based on (ZrO_2_)_0.99−x_(Sc_2_O_3_)_x_(R_2_O_3_)_0.01_ (R—Yb, Y, Tb, Gd) solid solutions was studied.

Four series of crystals were grown by the method of directional crystallization of the melt in a cold crucible, namely, (ZrO_2_)_0.99−x_(Sc_2_O_3_)_x_(Yb_2_O_3_)_0.01_, (ZrO_2_)_0.99−x_(Sc_2_O_3_)_x_(Tb_2_O_3_)_0.01_, (ZrO_2_)_0.99−x_(Sc_2_O_3_)_x_(Y_2_O_3_)_0.01_, and (ZrO_2_)_0.99−x_(Sc_2_O_3_)_x_(Gd_2_O_3_)_0.01_. The grown crystals were annealed in an air atmosphere at a temperature of 1000 °C for 400 h.

The appearance of the crystals, depending on the composition, varied from completely transparent crystals to completely muddy, as well as optically inhomogeneous crystals. The study of crystals by X-ray diffractometry showed the presence of tetragonal, cubic and rhombohedral modifications of zirconium dioxide, the presence and content of which depended on the concentration of Sc_2_O_3_ and the type of rare earth oxide. Thus, crystals containing 8 mol% Sc_2_O_3_ were a tetragonal modification of zirconium dioxide in the entire volume of crystals, regardless of the type of stabilizing oxide. It has been shown that the minimum Sc_2_O_3_ concentration required to stabilize the cubic phase is 9 mol% in the case of codoping with Yb_2_O_3_ or Tb_2_O_3_ and 10 mol% for Y_2_O_3_ or Gd_2_O_3_. The crystal lattice parameters differed depending on the type of rare earth oxide cation, namely, they increased in the series Yb < Tb < Y < Gd, which is in good agreement with the increase in the ionic radius of the stabilizing oxide cation. After annealing, all single-phase crystals (both tetragonal and cubic) remained single phase. The annealing of tetragonal crystals led to an increase in their degree of tetragonality, while the lattice parameters of the cubic phase did not change. The Raman spectroscopy technique in the spectra of the cubic crystals showed bands characteristic of the cubic phase, which is attributed to the presence of the pseudocubic t” phase in the crystals. The study of the local structure of the crystals by optical spectroscopy did not reveal any changes in the optical centers after annealing. The study of the specific conductivity of the crystals by impedance spectroscopy showed that the maximum values of electrical conductivity in each series of crystals were observed for single-phase pseudocubic (t”) solid solutions. Among these samples, the conductivity decreased in the series 9Sc1YbSZ > 9Sc1TbSZ > 10Sc1TbSZ > 10Sc1YSZ > 10Sc1GdSZ. This sequence correlates with a change in the lattice parameter of cubic crystals, which increases with an increase in the ionic radius of the rare earth cation. The annealing of single-phase pseudocubic crystals did not lead to noticeable changes in electrical conductivity. The conductivity degradation during annealing was observed only for tetragonal and two-phase crystals.

## Figures and Tables

**Table 1:** Chemical and phase compositions of grown crystals, their designations, and appearance characteristics.

Chemical Composition	Short Designation	Appearance		Phase Composition	
		After Growth	After Annealing	After Growth	After Annealing
**(ZrO_2_)_0.99−x_(Sc_2_O_3_)_x_(Yb_2_O_3_)_0.01_ series**					
(ZrO_2_)_0.91_(Sc_2_O_3_)_0.08_(Yb_2_O_3_)_0.01_	8Sc1YbSZ	muddy	muddy	t`	t`
(ZrO_2_)_0.90_(Sc_2_O3)_0.09_(Yb_2_O_3_)_0.01_	9Sc1YbSZ	transparent	transparent	c	c
(ZrO_2_)_0.89_(Sc_2_O_3_)_0.1_(Yb_2_O_3_)_0.01_	10Sc1YbSZ	inhomogeneous in volume (muddy and transparent areas)	muddy throughout	c + r	r
**(ZrO_2_)_0.99−x_(Sc_2_O_3_)_x_(Y_2_O_3_)_0.01_ series**					
(ZrO_2_)_0.91_(Sc_2_O_3_)_0.08_(Y_2_O_3_)_0.01_	8Sc1YSZ	muddy	muddy	t`	t`
(ZrO_2_)_0.90_(Sc_2_O_3_)_0.09_(Y_2_O_3_)_0.01_	9Sc1YSZ	inhomogeneous in volume (muddy and transparent areas)	inhomogeneous in volume (muddy and transparent areas)	t`+ c	t` + c
(ZrO_2_)_0.89_(Sc_2_O_3_)_0.1_(Y_2_O_3_)_0.01_	10Sc1YSZ	transparent	transparent	c	c
**(ZrO_2_)_0.99−x_(Sc_2_O_3_)_x_(Tb_2_O_3_)_0.01_ series**					
ZrO_2_)_0.91_(Sc_2_O_3_)_0.08_(Tb_2_O_3_)_0.01_	8Sc1TbSZ	muddy, yellow color	muddy, yellow color	t`	t`
(ZrO_2_)_0.90_(Sc_2_O_3_)_0.09_(Tb_2_O_3_)_0.01_	9Sc1TbSZ	transparent, yellow color	transparent, yellow color	c	c
(ZrO_2_)_0.89_(Sc_2_O_3_)_0.10_(Tb_2_O_3_)_0.01_	10Sc1TbSZ	transparent, yellow color	transparent, yellow color	c	c
**(ZrO_2_)_0.99−x_(Sc_2_O_3_)_x_(Gd_2_O_3_)_0.01_ series**					
(ZrO_2_)_0.89_ (Sc_2_O_3_)_0.08_(Gd_2_O_3_)_0.01_	8Sc1GdSZ	muddy	muddy	t`	t`
(ZrO_2_)_0.90_ (Sc_2_O_3_)_0.09_(Gd_2_O_3_)_0.01_	9Sc1GdSZ	inhomogeneous in volume (muddy and transparent areas)	muddy	t` + c	t`
(ZrO_2_)_0.89_(Sc_2_O_3_)_0.1_(Gd_2_O_3_)_0.01_	10Sc1GdSZ	transparent	transparent	c	c

t`—tetragonal phase with a low degree of tetragonality; c—cubic phase; r—rhombohedral phase.

**Table 2:** Lattice parameters of the tetragonal phase of crystals containing 8 mol% Sc_2_O_3_ before and after annealing.

Type of Stabilizing Oxide	Lattice Parameter *a*, nm		Lattice Parameter *c*, nm	
	Before Annealing	After Annealing	Before Annealing	After Annealing
Yb_2_O_3_	0.35982 (1)	0.35981 (1)	0.51131 (2)	0.51143 (2)
Tb_2_O_3.5_	0.35993 (1)	0.35992 (1)	0.51138 (2)	0.51147 (2)
Y_2_O_3_	0.36004 (1)	0.36004 (1)	0.51190 (2)	0.51211 (2)
Gd_2_O_3_	0.36017 (1)	0.36016 (1)	0.51198 (2)	0.51219 (2)

**Table 3:** Lattice parameters of the cubic phase before and after crystal annealing.

Type of Stabilizing Oxide	Lattice Parameter *a*, nm			
	9 mol% Sc_2_O_3_		10 mol% Sc_2_O_3_	
	Before Annealing	After Annealing	Before Annealing	After Annealing
Yb_2_O_3_	0.50937 (1)	0.50937 (1)	0.50931 (1)	
Tb_2_O_3.5_	0.50955 (1)	0.50950 (1)	0.50943 (1)	0.50938 (1)
Y_2_O_3_	0.50962 (1)	0.50962 (1)	0.50959 (1)	0.50958 (1)
Gd_2_O_3_	0.50982 (1)	0.50983 (1)	0.50973 (1)	0.50973 (1)

## Data Availability

All the data are available within the manuscript.
